# Antimicrobial Photodynamic Coatings Reduce the Microbial Burden on Environmental Surfaces in Public Transportation—A Field Study in Buses

**DOI:** 10.3390/ijerph19042325

**Published:** 2022-02-17

**Authors:** Larissa Kalb, Pauline Bäßler, Wulf Schneider-Brachert, Daniel Bernhard Eckl

**Affiliations:** 1 Department of Dermatology, University Hospital Regensburg, 93053 Regensburg, Germany; larissa.kalb@klinik.uni-regensburg.de (L.K.); pauline.baessler@klinik.uni-regensburg.de (P.B.); 2 Department of Infection Control and Infectious Diseases, University Hospital, 93053 Regensburg, Germany; wulf.schneider@klinik.uni-regensburg.de; 3 Department of Microbiology, University of Regensburg, 93053 Regensburg, Germany

**Keywords:** antimicrobial coating, photodynamic inactivation, public transportation, AMC

## Abstract

Millions of people use public transportation daily worldwide and frequently touch surfaces, thereby producing a reservoir of microorganisms on surfaces increasing the risk of transmission. Constant occupation makes sufficient cleaning difficult to achieve. Thus, an autonomous, permanent, antimicrobial coating (AMC) could keep down the microbial burden on such surfaces. A photodynamic AMC was applied to frequently touched surfaces in buses. The microbial burden (colony forming units, cfu) was determined weekly and compared to equivalent surfaces in buses without AMC (references). The microbial burden ranged from 0–209 cfu/cm^2^ on references and from 0–54 cfu/cm^2^ on AMC. The means were 13.4 ± 29.6 cfu/cm^2^ on references and 4.5 ± 8.4 cfu/cm^2^ on AMC (*p* < 0.001). The difference in microbial burden on AMC and references was almost constant throughout the study. Considering a hygiene benchmark of 5 cfu/cm^2^, the data yield an absolute risk reduction of 22.6% and a relative risk reduction of 50.7%. In conclusion, photodynamic AMC kept down the microbial burden, reducing the risk of transmission of microorganisms. AMC permanently and autonomously contributes to hygienic conditions on surfaces in public transportation. Photodynamic AMC therefore are suitable for reducing the microbial load and closing hygiene gaps in public transportation.

## 1. Introduction

Microbes are present not only on surfaces in hospitals but also in all public areas including public transportation. In the Journal *Nature*, Rachel Ehrenberg mentioned a survey of genetic material from surfaces in the New York City’s subway system that found more than 1000 taxa, of which half were known and half of them were unknown [1,2]. More recently, researchers from all over the world created a global atlas containing 4728 metagenomic samples from mass-transit systems in 60 cities over 3 years [3]. The study identified the most common antimicrobial resistance (AMR) genes among the metagenomic samples, which refer to antibiotics such as macrolides, lincosamides, streptogramins and β-lactams [3]. Another study by Panagiotou and colleagues demonstrated that surfaces in public transport systems in the Mass Transit Railway of Hong Kong contribute to the spreading of antimicrobial resistance genes (ARG). Most likely due to hand-to-surface transmission, bacteria with these ARG are disseminated among different lines. Particularly, ARG transmission for antibiotics such as tetracycline and vancomycin seems to be fostered by public transportation [4]. Furthermore, the same study as well as the mentioned global atlas showed that most of bacterial contamination on surfaces originated from human skin as the most abundant metagenomes are derived from skin commensals [3,4].

In the United States of America, people took almost 10 billion trips on public transportation in 2018, knowing that buses, subways and trains are—even macroscopically—not always the cleanest spaces [5]. In public transportation, people frequently touch different surfaces such as buttons, railings, handgrips and seats. Being constantly occupied throughout the day, sufficient cleaning inside vehicles is often difficult to achieve. Consequently, inanimate surfaces in public transportations are contaminated with microorganisms and viruses [5].

Unfortunately, only a few peer-reviewed studies are available quantifying the contamination of surfaces in public transportation with culture-based methods. An investigation on the public transport system and in public areas of a hospital in central London revealed a mean microbial burden on frequently touched surfaces of 12 colony-forming units per cm^2^ (cfu/cm^2^), whereas 8% of samples showed the presence of *S. aureus* [6]. A large-scale study on inanimate surfaces in different railway stations in England and Scotland revealed a bacterial contamination of up to 10^7^–10^8^ cfu/cm^2^, whereas the sampling sites were excluded from daily cleaning routine [7]. Another study from Bangladesh found a median microbial load of 3 × 10^5^ cfu/cm^2^ on handrails in public transport buses without cleaning [8]. Concerning the bacterial species, the metro in Mexico showed mainly *Cutibacterium*, *Corynebacterium* spp., *Streptococcus* spp., and *Staphylococcus* spp. [9], in New York city mainly *Enterobacter cloacae* [2] and *Acinetobacter* spp., and *Methylobacterium* in the subway of Barcelona [10]. When sampling surfaces in buses in Ohio, 17% of samples showed *S. aureus* [11].

Pathogenic bacteria, in particular antibiotic-resistant bacteria, represent a major threat to human health [12]. When using public transportation, the touch of contaminated surfaces is unavoidable. Germs of colonized surfaces can be transferred by hands to mouth or nose, possibly causing infections. Thus, the use of public transportation adds an additional risk of transmission of pathogens via contaminated surfaces, as also shown for hospitals [13,14].

To mitigate such a pathogen transmission, frequently touched inanimate surfaces can be equipped with an antimicrobial coating (AMC), initially developed to protect inanimate surfaces in health-care settings [15]. An AMC acts permanently and autonomously and can thereby reduce the contamination of surfaces complementing cleaning and hygiene procedures. Several AMC are known. Some are based on complex polymer substances such as parylene-c, poly-dimethyl siloxane, or poly-methyl methacrylate [16]. Others focus on the use of metal-based coatings such as zinc pyrithione [17,18], zinc oxide [19], copper [20], titan dioxide [21,22,23], or silver [24,25]. Biocides such as diuoron, and Sea-Nine 211 were also effective [17,26]. Additionally, there is a vast amount of enzyme-based coating alternatives [27]. Another noteworthy common chemical group used in antimicrobial coatings are so-called quaternary ammonium compounds or QACs [28,29]. Just recently, photodynamic coatings became of interest to researchers, mainly as they bear important advantages over other coatings. Since singlet oxygen is a gaseous molecule, it can easily leave the coating and reach the microorganisms directly on the surface via diffusion [30]. Consequently, singlet oxygen does not require any transport medium such as water but can kill bacteria on dry surfaces [15]. Contrary to biocides, the active substance of a photodynamically active coating is only present in a very thin layer above the surface, therefore it does not interfere significantly with the environment [30,31]. An AMC technology which is based on the photodynamic principle was already successfully tested in a field study in two hospitals [32]. This thin AMC coating contains a special molecule called photosensitizer that generates the so-called singlet oxygen when the coating is exposed to ambient, visible light. The gaseous singlet oxygen molecule can leave the AMC via diffusion, reaching the microorganisms present on the coated surface and killing them via oxidative damages [32]. The results of that field study proved the action of singlet oxygen, which killed bacteria on the coated surface and thereby significantly reduced the contamination.

In this study, the same technology was applied in buses of the local public transportation in Regensburg, Germany. The antimicrobial efficacy of the AMC was tested under highly demanding real-life conditions via sampling of coated and control surfaces inside the buses by culture-based methods.

## 2. Materials and Methods

**Figure 1:**
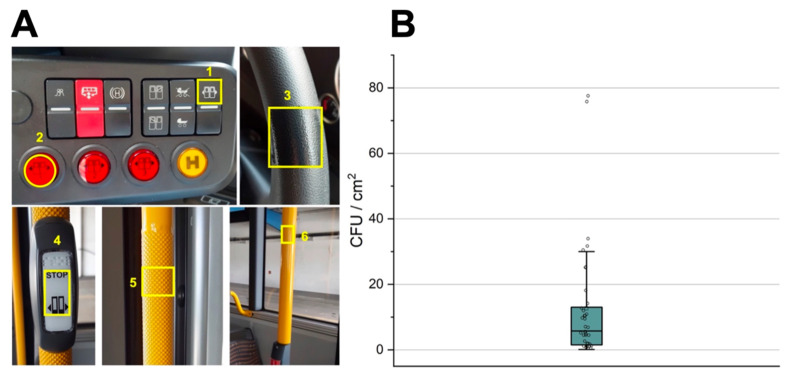
(**A**) Sampling sites of the public transport buses. Sites 1–3 were surfaces frequently touched by the operator including two door opening buttons (1, 2) and the steering wheel (3). Sampling sites 4–6 were frequently touched surfaces in the passenger part of the bus, including a stop button (4), a textured handrail (5) and a nontextured handrail (6). (**B**) The values of microbial burden of the prestudy sampling are shown as box plots with the median and the quartile ranges.

Four urban buses of Regensburg (das Stadtwerk Regensburg GmbH, Regensburg, Germany) were selected, showing comparable frequency of use. Six surfaces in all buses were exemplarily coated with DYPHOX^®^ technology (Dyphox, Regensburg, Germany), which should be frequently touched by passengers and/or bus drivers (Figure 1A). The used photosensitizer class absorbs light in the visible spectrum from 400–460 nm and is characterized by a singlet oxygen quantum yields close to unity. The permanent character of the coating was, on the one hand, demonstrated in another field study that was conducted over 6 months in two hospitals with the same coating [32]. On the other hand, the manufacturer of the coating commissioned an external lab that used a standard protocol according to artificial aging corresponding to 12 months of real-time aging. Afterwards, the external, independent lab measured the antimicrobial activity to exceed 99.99% bacterial reduction according to ISO 22196. The buses were on the road from 5 a.m. to 2 p.m., which made it possible to map the traffic load at the core commuting time, as the sampling took place immediately after the bus arrived at the terminal. 

Inanimate surfaces in two buses received photodynamic coatings of Dyphox^®^ universal coating, which is a retrofit application possible on various surface materials. The coating is applied as a liquid that rapidly hardens as a micron-thin, nearly invisible layer. The other two buses with the noncoated corresponding surfaces were used for sampling. The stability of all coatings was regularly checked each sampling day during the field study by a visual examination of the macroscopic integrity of the applied coating. The coating is certified to be resistant against several cleaning and disinfectant agents (EN ISO 2812-3). The antimicrobial efficacy of the coating has been successfully certified in a standardized 12-month aging test using ISO 22196.

**Laboratory tests.** Prior to the microbial sampling in the buses, the coating was validated concerning the antimicrobial activity in the laboratory. The coating was applied on a commercially available melamine resin-based laminate. *Staphylococcus aureus* (ATCC^®^ 25923^TM^) were resuspended in Millipore water + 0.1% Tween20. 50 µL of the suspension, approximately corresponding to 10^5^ colony forming units/mL, were placed on the coating, and kept in the dark until the samples were visibly dry. Afterwards, the samples were irradiated with a blue LED-based light source at a radiant exposure of 12 J/cm^2^. Dark controls and reference were kept in the dark for the same time. After light exposure, the bacteria were recovered using a sterile cotton-tipped swab that was resuspended in 0.9 % sodium chloride solution. A 10-fold dilution series was prepared and plated onto Mueller–Hinton agar plates which were incubated at 37 °C for 24 h. The colonies were counted and the cfu/mL value was calculated, followed up by a calculation of the logarithmic reduction referenced to a coated surface that did not contain photosensitizer. In total, 5 independent experiments were conducted and used to calculate means and standard deviation.

**Routine cleaning.** During the whole study, the routine cleaning procedures were left unchanged in the buses to avoid any potential bias on the study results. The buses were cleaned daily after the sampling was performed by first using a cleaning solution containing <5% anionic surfactants followed up by treatment with 70% (*v*/*v*) ethanol.

**Sampling and quantification of bacteria.** The samples were collected over two months in 2021. The evaluation of bacterial counts was based on the European standard EN 13697 [17,33]. All tested surfaces were sampled using a liquid-based collection and transport system (eSwab regular, Mast Diagnostica GmbH, Reinfeld, Germany) according to the manufacturer’s instructions. Collected samples were diluted in duplicates up to 10^−2^ and 500 µL of each dilution was plated on tryptone soy agar (TSA). Colonies were counted after 24 and 48 h, and only agar plates with a colony count of 14 to 330 were considered except for the undiluted sample. Counted values were converted into cfu/cm^2^ depending on the size of the respective sampled area. For sampling surface 1, an area of 4 cm^2^ was sampled, surface 2 had a total sampling surface of 3.14 cm^2^, the sample from surface 3 was obtained from an area of 12 cm^2^, surface 4 was sampled on 6 cm^2^, and surfaces 5 and 6 were sampled in a 16 cm^2^ area (Figure 1A). To ensure sufficient bacterial contamination of the surfaces to be tested, prior to the study itself the cfu/cm^2^ were evaluated on the same yet untreated surfaces over three days in two different buses.

**Statistical Analysis.** All bacterial counts are presented as median and mean ± SD. Values were compared between photodynamic coating and uncoated surface using the nonparametric Mann–Whitney-U test. Bacterial counts were further dichotomized using the cutoffs >5 cfu/cm^2^ and >2.5 cfu/cm^2^. The benchmarks were chosen according to the scientific literature [34,35,36,37,38]. Absolute and relative risk reductions for high counts of bacteria on surfaces as well as odds ratios were calculated as effect estimates. *p*-value < 0.05 was considered statistically significant. All analyses were performed using SPSS statistics software version 26.0.0 (IBM SPSS Software, Armonk, NY, USA).

## 3. Results

Prior to experiments with coated and uncoated surfaces, six samplings (*n* = 36) were performed in order to evaluate the surfaces in respect of their suitability for the actual study. The microbial burden on the six sampled surfaces ranged from 0.1 to 77.1 cfu/cm^2^. The median of the microbial burden was 5.75 cfu/cm^2^ and 12.6 ± 19.6 cfu/cm^2^ for the mean value (Figure 1B).

**Figure 2:**
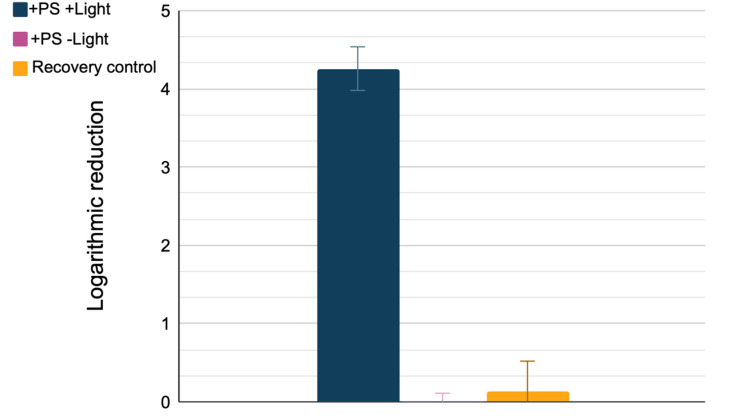
Results from laboratory experiments. The illuminated sample with photosensitizer in the coating achieves a logarithmic reduction exceeding 4 log_10_ steps (blue bar), while dark control (pink bar) and the recovery control (recovery control) led to no noteworthy bacterial reduction. Error bars represent the standard deviation of the experiments (*n* = 5).

The bacterial recovery with swabs from the coated surfaces in laboratory tests were excellent, with a loss equivalent to a bacterial reduction of 0.1 log_10_ steps (Figure 2). Laboratory tests further revealed that the surface had no detrimental dark toxicity effect with no measurable bacterial reduction compared to the reference sample (no light, no photosensitizer). Overall, under the given laboratory conditions, the tested coating led to a reduction of over 99.99 % of the bacteria (4.3 log_10_ steps). 

Overall, 14 samplings of the surfaces in the buses were performed at regular time intervals of one week to measure the bacterial counts on all included 24 surfaces of all buses. This yielded a total quantity of sample 336 bacterial samples, 168 on uncoated references and photodynamic coatings each.

**Figure 3:**
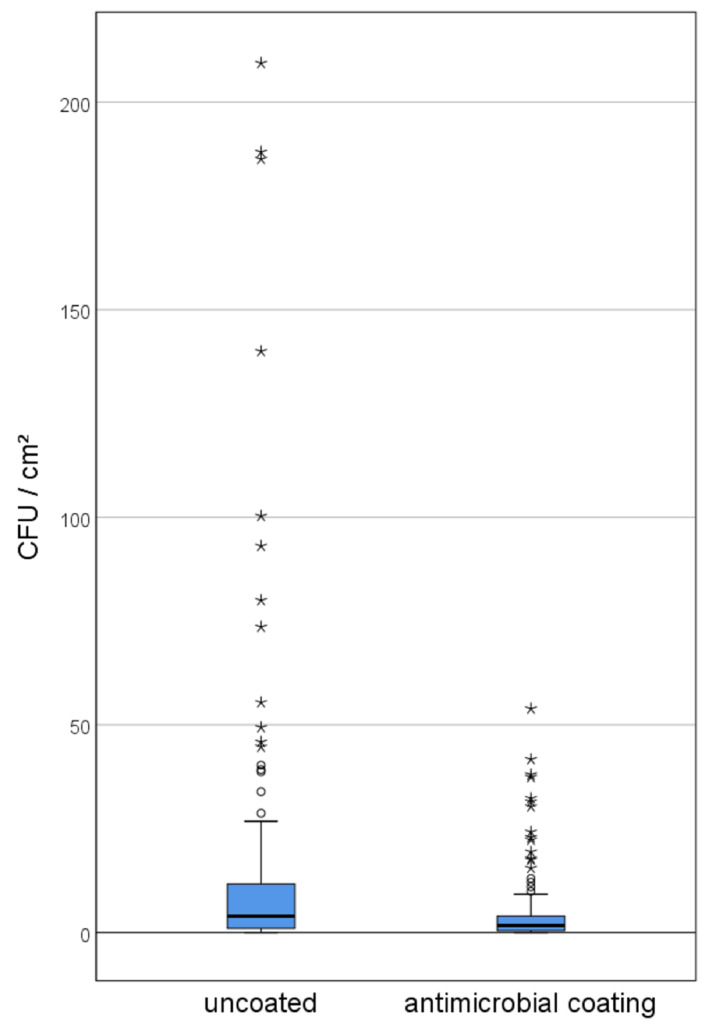
The values of microbial burden on uncoated references and on antimicrobial coatings are shown as box plots with the median and the quartile ranges.

The values of microbial burden ranged from 0–209 cfu/cm^2^ on uncoated references and 0–54 cfu/cm^2^ on sites with photodynamic coating. The median of bacterial burden on uncoated references or photodynamic coating was 3.8 or 1.8, respectively. The mean values of bacterial counts were 13.4 ± 29.6 cfu/cm^2^ on noncoated surfaces and 4.5 ± 8.4 cfu/cm^2^ on photodynamic AMC. The difference between mean values on photodynamic coating and noncoated surfaces was statistically significant with *p* < 0.001 (Figure 3). The obtained values obtained for the noncoated surface matched well with the results obtained from the initial sampling as mentioned above.

Bacterial counts were further dichotomized using the following cut-offs. The frequency of numbers with bacterial counts of >5 cfu/cm^2^ or >2.5 cfu/cm^2^ were significantly less on sites with AMC compared to uncoated references. When applying a benchmark of 5 cfu/cm^2^, the data yield an absolute risk reduction of 22.6% and a relative risk reduction of 50.7% for high bacteria counts on surface with an odds ratio of 0.35 (*p* < 0.001) (Table 1). Considering a benchmark of 2.5 cfu/cm^2^, the data yield an absolute risk reduction of 23.2% and a relative risk reduction of 39.0% for high bacteria counts on surface with an odds ratio of 0.39 (*p* < 0.001) (Table 1).

**Figure 4:**
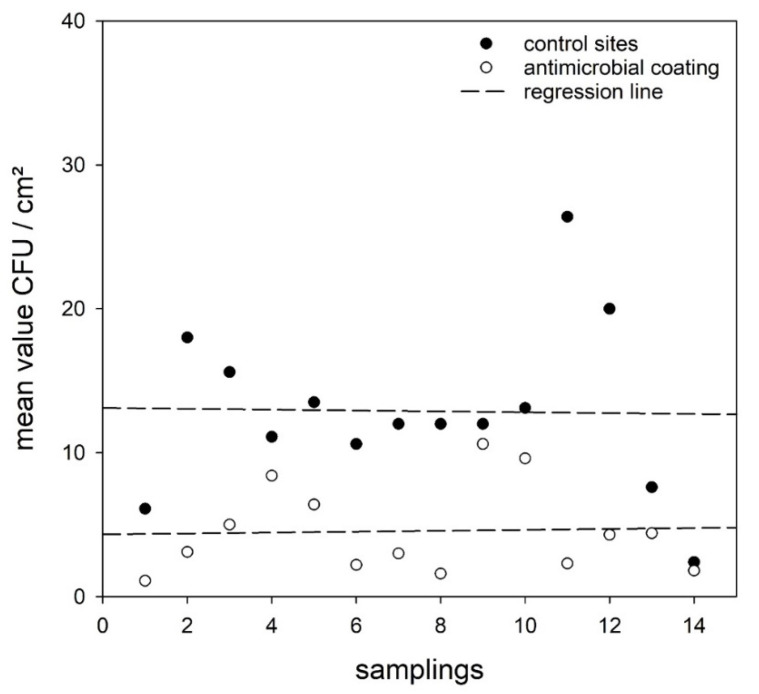
The mean values for all 14 samplings. The regression line indicates an almost constant difference between uncoated references without antimicrobial effect and the sites with the antimicrobial coating.

The mean values of microbial burden vary from sampling to sampling. However, the regression lines indicate an almost constant difference between the microbial burden found on uncoated references and photodynamic coating sites during the entire study (Figure 4).

## 4. Discussion

The present study clearly provided evidence that the photodynamic AMC can significantly decrease the bacterial burden on environmental surfaces in public transportation. Considering the study time of 2 months, the mean cfu value on the uncoated surface sites was 13.4 ± 29.6 cfu/cm^2^, comparable to 12 cfu/cm^2^ found on surfaces in other public transportation [6]. In contrast, the surfaces with photodynamic AMC showed a mean of 4.5 ± 8.4 cfu/cm^2^ only.

It is reasonable that the risk of microorganisms’ transmission should increase with the microbial burden on touched surfaces. Therefore, surfaces in hygiene-sensitive areas such as food industry and health-care settings should not exceed certain benchmarks, for instance 2.5 cfu/cm^2^ in hospitals [35,36] and 5 cfu/cm^2^ on food-processing equipment [37]. These internationally recognized figures can also serve as a starting point for environmental surfaces in public transportation. According to these proposed values, statistical analysis of our study data yielded a relative risk reduction of about 51% for high bacteria counts on surface when considering a benchmark of 5 cfu/cm^2^ (odds ratio 0.35, *p* < 0.001) (Table 1). Even for the smaller benchmark of 2.5 cfu/cm^2^, the relative risk reduction was 39% (odds ratio 0.38, *p* < 0.001) (Table 1) for high bacteria counts on surface.

The standard deviation of 29.6 cfu/cm^2^ on uncoated surfaces indicates a clear fluctuation of the respective microbial burden, ranging from 0–209 cfu/cm^2^. This should be nothing out of the ordinary, because the actual microbial burden depends on the touch frequency, the survival time of the germs, the cleaning cycles and the cleaning quality. Without cleaning or disinfection, the survival time of microorganisms on environmental surfaces ranges from hours to several months, and even viruses may stay infectious for up to several days [37,38]. Thus, regular sampling of the surfaces at fixed times should randomly detect the actual microbial burden.

In contrast to uncoated surfaces, the standard deviation was clearly smaller for the AMC (8.4 cfu/cm^2^) and the microbial burden ranged from 0–54 cfu/cm^2^ only. Thus, the photodynamic AMC can reduce the peak values of high bacterial counts and hence should reduce the risk of transmission through surfaces via the hands of any passenger or staff in public transportation. In addition, the regression analysis of the microbial burden on AMC and uncoated surfaces revealed that the AMC showed no decline in efficacy (Figure 4).

The microbial burden on surfaces in public areas coheres with the skin microbiome due to recurrent touches [4]. In addition, sneezing and coughing deposits microorganisms and viruses along with droplets on inanimate surfaces in the environment [39]. In health-care settings, clear evidence exist that contaminated human hands are a major vehicle for germs to inanimate surfaces [40,41]. Nevertheless, even in health care the compliance of hand hygiene and surface cleaning is not sufficient [42,43].

Good hand hygiene is also important in public areas, as it prevents the spread of diseases especially when travelling [44], studies suggest that 22% to 64% of international travelers become ill during or after travel [45]. However, the rates of hand washing in the general population is highly varying. For instance, among food workers, appropriate hand washing varied from 10 to 37% [46]. Well-executed hand hygiene and compliance can reduce the risk of diarrheal disease transmission by between 23% and 48% [47,48].

A self-reported survey among the general population in the UK reported that only 61% wash their hands after toilet use [45]. A comparable study investigated the compliance rate of hand washing in Germany, and women reported ‘almost always’ with a compliance of 30.8% (men: 20.3%) in situations such as public restroom visits [49], but one should consider the weaknesses of self-reported compliance compared to observational studies [45]. Currently, the situation might have improved due to the COVID-19 pandemic. A study in 2020 showed that approximately 85% of U.S. adults are frequently engaged in handwashing or using hand sanitizer after contact with high-touch public surfaces, including only 72.4% of those aged 18–24 years [50]. It was shown elsewhere that such a high hand hygiene compliance, induced by a pandemic, can decrease over time when the fear of infection with SARS-CoV-2 decreased [51].

In the light of the low compliance rates of hand hygiene, the presence of microbes on inanimate surfaces in public transportation is unavoidable. Cleaning or disinfection of all these numerous surfaces in buses, subways and trains seems to be a giant undertaking that would require substantial human resources and logistics. In addition, the disinfection of a surface acts only at the time of its execution, and recontamination inevitably occurs. 

Thus, AMC can complement routine cleaning and disinfection procedures. AMC act independently and autonomously without assistance of the staff, in particular in the time gap of routine disinfections. This produces a permanent reduction in the mean number of microbes on such coated surfaces, thereby reducing the risk of transmission [15]. 

The antimicrobial efficacy of photodynamic AMC was already proven in laboratory experiments, showing up to 4 log_10_ of bacterial reduction for bacteria such as *S. aureus* [32]. Such laboratory tests are necessary to conduct research and development linked to AMC. However, laboratory tests are intrinsically artificial as it is impossible to mimic real life conditions in laboratories [15]. Thus, testing the efficacy of AMC should be mandatory in the subsequent field of application. The photodynamic AMC was recently tested in a field study in two hospitals on different surfaces for several months, revealing that the photodynamic AMC reduced the mean cfu/cm^2^ and its standard deviation statistically significantly [32]. 

At present, different technologies exist to equip inanimate surfaces with AMC, which may contain biocidal substances such as silver, copper, titanium dioxide or quaternary ammoniums. Many of these AMC technologies were tested under laboratory conditions only, in particular with procedures using wet conditions only such as ISO 22196 (bacteria) or ISO 21702 (virus) [15,52,53]. In case of silver AMC, it has already been shown that the antimicrobial efficacy disappeared when surfaces are dry, as in reality under normal ambient humidity conditions [54]. This is not surprising, as most of the AMC biocides need a liquid on the inanimate surface for transportation of the biocidal substance from the AMC to the microbes. In addition, most laboratory tests were performed on thoroughly cleaned AMC, because soiling hampers the antimicrobial efficacy, in particular for copper and quaternary ammoniums [15].

The photodynamic approach is effective on normal dry surfaces because the biocidal singlet oxygen is a gaseous molecule, escaping the AMC after generation by the photosensitizer. Any soiling present on surfaces during the study obviously did not hamper the antimicrobial efficacy, as shown by the significant reduction in microbial burden on the photodynamic AMC.

Another advantage of singlet oxygen is the fact that this molecule approaches the microbial cells from outside and oxidizes any double bonds in the microbial envelope. The cells are damaged without the need for penetration of singlet oxygen into the cells. Therefore, the photodynamic approach is considered to provoke no resistance in microorganisms [55]. 

This is an important issue when AMC technologies will be used on surfaces in public areas including public transport. Various microorganisms already show reduced sensitivity or resistance to biocidal substances such as chlorhexidine, triclosan, silver, copper, and quaternary ammonium compounds [56,57]. In 2021, the European Medical Association stated that metals such as copper, zinc and silver, which were used in AMC, are also known to elicit coselection for AMR genes and thus might play a role in the development and spread of AMR [58]. The co- or cross-resistance induced by such metals may lead to bacteria that also show an increased resistance to antibiotics [15,59]. 

Biocidal substances with a long-term stability such as metals and quaternary ammonium may also reach the environment, and thereby for example influence wastewater treatment plants in a detrimental manner [60]. In addition, EU authorities demand safe chemicals while preventing harm to humans and the environment by avoiding substances of concern for nonessential use [61]. Singlet oxygen is short-lived and therefore remains active only in a thin layer of a few millimeters above AMC and cannot accumulate in the environment [31]. Singlet oxygen has been safely applied in medicine, such as for photodynamic treatment of tumors and age-related macular disease of the retina, for many years [62,63].

## 5. Conclusions

The photodynamic AMC significantly reduced the mean bacterial burden on different inanimate surfaces in urban buses under real-life conditions. In addition, the maximal detected bacterial burden (cfu/cm^2^) was clearly reduced by photodynamic AMC, as shown by the reduced standard deviation. Photodynamic AMC are therefore not only suitable for reducing the microbial load in health-care-associated environments but also for closing hygiene gaps in public transportation.

## Figures and Tables

**Table 1:** Microbial burden regarding different benchmarks. The numbers given are the events concerning the given benchmark.

Benchmarks	Uncoated (*n* = 168)		Antimicrobial Coating (*n* = 168)	
	Number	Percent	Number	Percent
cfu/cm^2^ ≤ 2.5	68	40.5%	107	63.7%
cfu/cm^2^ > 2.5	100	59.5%	61	36.3%
cfu/cm^2^ ≤ 5	93	55.4%	131	78.0%
cfu/cm^2^ > 5	75	44.6%	37	22.0%

## Data Availability

Data are contained within the article.

## References

[1] Ehrenberg R. (2015). Urban microbes come out of the shadows. Nat. News.

[2] Afshinnekoo E., Meydan C., Chowdhury S., Jaroudi D., Boyer C., Bernstein N., Maritz J.M., Reeves D., Gandara J., Chhangawala S. (2015). Geospatial resolution of human and bacterial diversity with city-scale metagenomics. Cell Syst..

[3] Danko D., Bezdan D., Afshin E.E., Ahsanuddin S., Bhattacharya C., Butler D.J., Chng K.R., Donnellan D., Hecht J., Jackson K. (2021). A global metagenomic map of urban microbiomes and antimicrobial resistance. Cell.

[4] Kang K., Ni Y., Li J., Imamovic L., Sarkar C., Kobler M.D., Heshiki Y., Zheng T., Kumari S., Wong J.C.Y. (2018). The environmental exposures and inner-and intercity traffic flows of the metro system may contribute to the skin microbiome and resistome. Cell Rep..

[5] Costello C. Are “Bad” Microbes Getting a Free Ride on Your Transit System?. https://www.metro-magazine.com/10111889/are-bad-microbes-getting-a-free-ride-on-your-transit-system.

[6] Otter J.A., French G.L. (2009). Bacterial contamination on touch surfaces in the public transport system and in public areas of a hospital in London. Lett. Appl. Microbiol..

[7] Patel K.V., Bailey C.L., Harding A., Biggin M., Crook B. (2018). Background levels of micro-organisms in the busy urban environment of transport hubs. J. Appl. Microbiol..

[8] Chowdhury T., Mahmud A., Barua A., Khalil M.D.I., Chowdhury R., Ahamed F., Dhar K. (2016). Bacterial contamination on hand touch surfaces of public buses in Chittagong city, Bangladesh. J. Environ. Sci. Toxicol. Food Technol..

[9] Hernández A.M., Vargas-Robles D., Alcaraz L.D., Peimbert M. (2020). Station and train surface microbiomes of Mexico City’s metro (subway/underground). Sci. Rep..

[10] Triadó-Margarit X., Veillette M., Duchaine C., Talbot M., Amato F., Minguillón M.C., Martins V., de Miguel E., Casamayor E.O., Moreno T. (2017). Bioaerosols in the Barcelona subway system. Indoor Air.

[11] Lutz J.K. (2011). Methicillin-Resistant Staphylococcus aureus on Public Transportation Vehicles: Sampler Performance, Prevalence, and Epidemiology. Ph.D. Thesis.

[12] Turner N.A., Sharma-Kuinkel B.K., Maskarinec S.A., Eichenberger E.M., Shah P.P., Carugati M., Holland T.L., Fowler V.G. (2019). Methicillin-resistant Staphylococcus aureus: An overview of basic and clinical research. Nat. Rev. Microbiol..

[13] Kanamori H., Rutala W.A., Weber D.J. (2017). The role of patient care items as a fomite in healthcare-associated outbreaks and infection prevention. Clin. Infect. Dis..

[14] Correa-Martinez C.L., Tönnies H., Froböse N.J., Mellmann A., Kampmeier S. (2020). Transmission of vancomycin-resistant enterococci in the hospital setting: Uncovering the patient-environment interplay. Microorganisms.

[15] Bäumler W., Eckl D., Holzmann T., Schneider-Brachert W. (2021). Antimicrobial coatings for environmental surfaces in hospitals: A potential new pillar for prevention strategies in hygiene. Crit. Rev. Microbiol..

[16] Mariello M., Guido F., Mastronardi V.M., Giannuzzi R., Algieri L., Qualteri A., Maffezzoli A., De Vittorio M. (2019). Reliability of Protective Coatings for Flexible Piezoelectric Transducers in Aqueous Environments. Micromachines.

[17] Yebra D.M., Kiil S., Dam-Johansen K. (2004). Antifouling technology—Past, present and future steps towards efficient and environmentally friendly antifouling coatings. Prog. Org. Coat..

[18] Pittol M., Tomacheski D., Simões D.N., Ribeiro V.F., Santana R.M.C. (2017). Antimicrobial performance of thermoplastic elastomers containing zinc pyrithione and silver nanoparticles. Mater. Res..

[19] Martinaga Pintarić L., Somogi Škoc M., Ljoljić Bilić V., Pokrovac I., Kosalec I., Rezić I. (2020). Synthesis, modification and characterization of antimicrobial textile surface containing ZnO nanoparticles. Polymers.

[20] Thukkaram M., Vaidulych M., Kylián O., Rigole P., Aliakbarshirazi S., Asadian M., Nikiforov A., Biederman H., Coenye T., Du Laing G. (2021). Biological activity and antimicrobial property of Cu/aC: H nanocomposites and nanolayered coatings on titanium substrates. Mater. Sci. Eng. C.

[21] Nakano R., Hara M., Ishiguro H., Yao Y., Ochiai T., Nakata K., Murakami T., Kajioka J., Sunada K., Hashimoto K. (2013). Broad spectrum microbicidal activity of photocatalysis by TiO_2_. Catalysts.

[22] Fisher L., Ostovapour S., Kelly P., Whitehead K.A., Cooke K., Storgårds E., Verran J. (2014). Molybdenum doped titanium dioxide photocatalytic coatings for use as hygienic surfaces: The effect of soiling on antimicrobial activity. Biofouling.

[23] Li R., Jin T.Z., Liu Z., Liu L. (2018). Antimicrobial double-layer coating prepared from pure or doped-titanium dioxide and binders. Coatings.

[24] Varghese S., Elfakhri S., Sheel D.W., Sheel P., Bolton F.J., Foster H.A. (2013). Novel antibacterial silver-silica surface coatings prepared by chemical vapour deposition for infection control. J. Appl. Microbiol..

[25] Scuri S., Petrelli F., Grappasonni I., Idemudia L., Marchetti F., Di Nicola C. (2019). Evaluation of the antimicrobial activity of novel composite plastics containing two silver (I) additives, acyl pyrazolonate and acyl pyrazolone. Acta Bio Med. Atenei Parm..

[26] Fay F., Linossier I., Langlois V., Vallee-Rehel K., Krasko M.Y., Domb A.J. (2007). Protecting biodegradable coatings releasing antimicrobial agents. J. Appl. Polym. Sci..

[27] Olsen S.M., Pedersen L.T., Laursen M.H., Kiil S., Dam-Johansen K. (2007). Enzyme-based antifouling coatings: A review. Biofouling.

[28] Druvari D., Koromilas N.D., Lainioti G.C., Bokias G., Vasilopoulos G., Vantarakis A., Baras I., Dourala N., Kallitsis J.K. (2016). Polymeric quaternary ammonium-containing coatings with potential dual contact-based and release-based antimicrobial activity. ACS Appl. Mater. Interfaces.

[29] Bieser A.M., Tiller J.C. (2011). Mechanistic considerations on contact-active antimicrobial surfaces with controlled functional group densities. Macromol. Biosci..

[30] Felgenträger A., Maisch T., Späth A., Schröder J.A., Bäumler W. (2014). Singlet oxygen generation in porphyrin-doped polymeric surface coating enables antimicrobial effects on Staphylococcus aureus. Phys. Chem. Chem. Phys..

[31] Wang K.-K., Song S., Jung S.-J., Hwang J.-W., Kim M.-G., Kim J.-H., Sung J., Lee J.-K., Kim Y.-R. (2020). Lifetime and diffusion distance of singlet oxygen in air under everyday atmospheric conditions. Phys. Chem. Chem. Phys..

[32] Eichner A., Holzmann T., Eckl D.B., Zeman F., Koller M., Huber M., Pemmerl S., Schneider-Brachert W., Bäumler W. (2020). Novel photodynamic coating reduces the bioburden on near-patient surfaces thereby reducing the risk for onward pathogen transmission: A field study in two hospitals. J. Hosp. Infect..

[33] (2019). Chemische Desinfektionsmittel und Antiseptika—Quantitativer Oberflächen-Versuch zur Bestimmung der bakteriziden und/oder fungiziden Wirkung chemischer Desinfektionsmittel auf nicht porösen Oberflächen in den Bereichen Lebensmittel, Industrie, Haushalt u.

[34] Dancer S.J. (2014). Controlling hospital-acquired infection: Focus on the role of the environment and new technologies for decontamination. Clin. Microbiol. Rev..

[35] White L.F., Dancer S.J., Robertson C., McDonald J. (2008). Are hygiene standards useful in assessing infection risk?. Am. J. Infect. Control.

[36] Dancer S.J. (2004). How do we assess hospital cleaning? A proposal for microbiological standards for surface hygiene in hospitals. J. Hosp. Infect..

[37] Kramer A., Assadian O. (2014). Survival of microorganisms on inanimate surfaces. Use of Biocidal Surfaces for Reduction of Healthcare Acquired Infections.

[38] Van Doremalen N., Bushmaker T., Morris D.H., Holbrook M.G., Gamble A., Williamson B.N., Tamin A., Harcourt J.L., Thornburg N.J., Gerber S.I. (2020). Aerosol and surface stability of SARS-CoV-2 as compared with SARS-CoV-1. N. Engl. J. Med..

[39] Dhand R., Li J. (2020). Coughs and sneezes: Their role in transmission of respiratory viral infections, including SARS-CoV-2. Am. J. Respir. Crit. Care Med..

[40] Chowdhury D., Tahir S., Legge M., Hu H., Prvan T., Johani K., Whiteley G.S., Glasbey T.O., Deva A.K., Vickery K. (2018). Transfer of dry surface biofilm in the healthcare environment: The role of healthcare workers’ hands as vehicles. J. Hosp. Infect..

[41] Suleyman G., Alangaden G., Bardossy A.C. (2018). The role of environmental contamination in the transmission of nosocomial pathogens and healthcare-associated infections. Curr. Infect. Dis. Rep..

[42] Carling P.C., Parry M.M., Rupp M.E., Po J.L., Dick B., Von Beheren S., Group H.E.H.S. (2008). Improving cleaning of the environment surrounding patients in 36 acute care hospitals. Infect. Control Hosp. Epidemiol..

[43] Clancy C., Delungahawatta T., Dunne C.P. (2021). Hand hygiene-related clinical trials reported between 2014 and 2020: A comprehensive systematic review. J. Hosp. Infect..

[44] Shaban R.Z., Sotomayor-Castillo C.F., Malik J., Li C. (2020). Global commercial passenger airlines and travel health information regarding infection control and the prevention of infectious disease: What’s in a website?. Travel Med. Infect. Dis..

[45] Lawson A., Vaganay-Miller M., Cameron R. (2021). An Investigation of the General Population’s Self-Reported Hand Hygiene Behaviour and Compliance in a Cross-European Setting. Int. J. Environ. Res. Public Health.

[46] Green L.R., Selman C.A., Radke V., Ripley D., Mack J.C., Reimann D.W., Stigger T., Motsinger M., Bushnell L. (2006). Food worker hand washing practices: An observation study. J. Food Prot..

[47] Cairncross S., Hunt C., Boisson S., Bostoen K., Curtis V., Fung I.C.H., Schmidt W.-P. (2010). Water, sanitation and hygiene for the prevention of diarrhoea. Int. J. Epidemiol..

[48] Freeman M.C., Stocks M.E., Cumming O., Jeandron A., Higgins J.P.T., Wolf J., Prüss-Ustün A., Bonjour S., Hunter P.R., Fewtrell L. (2014). Systematic review: Hygiene and health: Systematic review of handwashing practices worldwide and update of health effects. Trop. Med. Int. Health.

[49] Mardiko A.A., von Lengerke T. (2020). When, how, and how long do adults in Germany self-reportedly wash their hands? Compliance indices based on handwashing frequency, technique, and duration from a cross-sectional representative survey. Int. J. Hyg. Environ. Health.

[50] Czeisler M.É., Garcia-Williams A.G., Molinari N.-A., Gharpure R., Li Y., Barrett C.E., Robbins R., Facer-Childs E.R., Barger L.K., Czeisler C.A. (2020). Demographic Characteristics, Experiences, and Beliefs Associated with Hand Hygiene Among Adults During the COVID-19 Pandemic—United States, June 24–30, 2020. Morb. Mortal. Wkly. Rep..

[51] Makhni S., Umscheid C.A., Soo J., Chu V., Bartlett A., Landon E., Marrs R. (2021). Hand Hygiene Compliance Rate During the COVID-19 Pandemic. JAMA Intern. Med..

[52] (2011). Measurement of Antibacterial Activity on Plastics and Other Non-Porous Surfaces.

[53] (2019). Measurement of Antiviral Activity on Plastics and Other Non-Porous Surfaces.

[54] Michels H.T., Noyce J.O., Keevil C.W. (2009). Effects of temperature and humidity on the efficacy of methicillin-resistant Staphylococcus aureus challenged antimicrobial materials containing silver and copper. Lett. Appl. Microbiol..

[55] Wainwright M., Maisch T., Nonell S., Plaetzer K., Almeida A., Tegos G.P., Hamblin M.R. (2017). Photoantimicrobials—Are we afraid of the light?. Lancet Infect. Dis..

[56] Alquethamy S.F., Adams F.G., Naidu V., Khorvash M., Pederick V.G., Zang M., Paton J.C., Paulsen I.T., Hassan K.A., Cain A.K. (2019). The role of zinc efflux during Acinetobacter baumannii infection. ACS Infect. Dis..

[57] Williamson D.A., Carter G.P., Howden B.P. (2017). Current and emerging topical antibacterials and antiseptics: Agents, action, and resistance patterns. Clin. Microbiol. Rev..

[58] EMA Reflection Paper on Antimicrobial Resistance in the Environment. https://www.ema.europa.eu/en/documents/scientific-guideline/reflection-paper-antimicrobial-resistance-environment-considerations-current-future-risk-assessment_en.pdf.

[59] Pal C., Asiani K., Arya S., Rensing C., Stekel D.J., Larsson D.G.J., Hobman J.L. (2017). Metal resistance and its association with antibiotic resistance. Adv. Microb. Physiol..

[60] Zhang X., Ma J., Chen M., Wu Z., Wang Z. (2018). Microbial responses to transient shock loads of quaternary ammonium compounds with different length of alkyl chain in a membrane bioreactor. AMB Express.

[61] European Commission Chemicals Strategy for Sustainability towards a Toxic-Free Environment. https://ec.europa.eu/environment/pdf/chemicals/2020/10/Strategy.pdf.

[62] Gunaydin G., Gedik M.E., Ayan S. (2021). Photodynamic Therapy for the Treatment and Diagnosis of Cancer—A Review of the Current Clinical Status. Front. Chem..

[63] Nicolò M., Ferro Desideri L., Vagge A., Traverso C.E. (2020). Current pharmacological treatment options for central serous chorioretinopathy: A review. Pharmaceuticals.

